# From Iron Deficiency to Overload: A Missing Link in the Mechanisms of Cardiac Autonomic Nervous System Dysfunction

**DOI:** 10.3390/jcm15051871

**Published:** 2026-02-28

**Authors:** Krzysztof Młodziński, Michał Świątczak, Damian Kaufmann, Klaudia Rybka, Jacek Wolf, Ludmiła Daniłowicz-Szymanowicz

**Affiliations:** 1 II Department of Cardiology and Electrotherapy, Medical University of Gdańsk, Dębinki 7, 80-211 Gdańsk, Poland; krzysztofmlodzinski@gumed.edu.pl (K.M.);; 2 Department of Hypertension and Diabetology, Medical University of Gdańsk, Dębinki 7, 80-211 Gdańsk, Poland

**Keywords:** autonomic nervous system dysfunction, iron metabolism disorder, iron overload, iron deficiency, hereditary hemochromatosis, beta-thalassemia, sickle cell anemia

## Abstract

The autonomic nervous system (ANS) plays a key role in cardiovascular regulation by maintaining hemodynamic and metabolic homeostasis through balanced sympathetic and parasympathetic activity. While autonomic dysfunction is classically associated with diabetes, neurodegenerative diseases, autoimmune neuropathies, and chronic cardiovascular conditions, growing evidence suggests that disturbances in iron metabolism represent an underrecognized contributor to cardiac autonomic dysregulation. This narrative review summarizes data from 107 studies on ANS disorders, including 49 investigating cardiovascular involvement. Reported abnormalities included reduced heart rate variability and baroreflex sensitivity, prolonged P-wave duration and QT dispersion, and deviations in non-invasive autonomic testing parameters. In iron overload states, these changes appear to be driven primarily by oxidative stress, whereas in iron deficiency they are likely mediated by tissue hypoxia. Importantly, several studies indicate that normalization of iron homeostasis may partially reverse autonomic dysfunction. This potentially reversible component underscores the clinical relevance of screening for and correcting iron imbalance not only to improve hematological status but also to reduce cardiovascular risk. Large-scale, multicenter studies using standardized autonomic assessment protocols are required to clarify prognostic implications and inform evidence-based clinical guidelines.

## 1. Introduction

The autonomic nervous system (ANS) plays a vital role in maintaining cardiovascular, metabolic, and overall homeostatic balance through coordinated interactions between its sympathetic and parasympathetic branches. Dysfunction of the ANS manifests clinically as a broad spectrum of symptoms, including orthostatic hypotension, resting tachycardia, gastrointestinal dysmotility, abnormal sweating, bladder dysfunction, and impaired thermoregulation. These manifestations reflect multisystem involvement and frequently coexist with fatigue, exercise intolerance, and sleep disturbances. Common etiologies of autonomic dysfunction included amyloidosis, diabetes mellitus, neurodegenerative disorders such as Parkinson’s disease and multiple system atrophy, autoimmune neuropathies, chronic kidney disease, and cardiovascular disorders. Both AL and transthyretin amyloidosis may lead to autonomic nervous system involvement, contributing to orthostatic hypotension, gastrointestinal dysmotility, and worsening heart failure (HF) symptoms [1]. In addition, subclinical autonomic imbalance is also increasingly recognized in metabolic and inflammatory conditions.

Iron is an essential micronutrient that supports a wide array of biological processes, including oxygen transport, cellular respiration and DNA synthesis and repair [2]. Owing to its redox activity, unbound iron can catalyze the formation of reactive oxygen species (ROS); therefore tight regulation of systemic and cellular iron levels is critical [3]. Dysregulation of iron metabolism could lead either to iron deficiency (ID) or iron overload (IO), each of which activates distinct yet partially overlapping, pathways of tissue injury. In IO disorders, such as hereditary hemochromatosis, β-thalassemia major, and sideroblastic anemia, the transferrin-binding capacity is exceeded, leading to accumulation of non-transferrin-bound iron (NTBI). NTBI readily enters cells via low-affinity pathways, forming labile iron pools that promote Fenton chemistry and excessive ROS generation. The resulting oxidative stress induce lipid peroxidation, protein oxidation, mitochondrial dysfunction, and ultimately cell death—mechanisms implicated in hepatic fibrosis, pancreatic β-cell loss, and myocardial injury [4]. Conversely, ID deprives cells of iron-dependent cofactors required for heme and iron-sulfur cluster enzymes synthesis, potentially impairing mitochondrial electron transport and antioxidant defenses [5]. Although NTBI is not generated in ID, disruption of iron-dependent enzymatic processes may indirectly increase ROS production and compromise cellular integrity [6].

Whether in deficiency or excess, iron imbalance exerts systemic effects that extend beyond classic target organs. Notably, ROS such as superoxide, peroxynitrite, and nitric oxide modulate cardiac autonomic signaling under physiological conditions, maintaining a balance between sympathetic stimulation against parasympathetic restraint [7]. Iron-driven ROS overproduction—amplified by angiotensin II–AT_1_ receptor activation in the rostral ventrolateral medulla, a key vasomotor center—may shift this balance toward sympathetic overactivity [8]. Experimental models of hypertension and HF demonstrate increased ROS levels, reduced antioxidant defenses (e.g., superoxide dismutase), and impaired nitric-oxide synthase coupling within autonomic nuclei, whereas antioxidant interventions attenuate sympathetic tone and improve baroreflex sensitivity (BRS) [9]. Collectively, these findings suggest that iron-mediated oxidative stress may represent a common mechanistic contributor to cardiac autonomic dysregulation across IO and ID states. Despite these emerging links, the influence of iron metabolism disorders on the autonomic nervous system remains insufficiently recognized and poorly characterized. Given the relevance of both IO and ID in modulating autonomic control, systematic synthesis of the available evidence is warranted to guide future research.

The aim of this review is to synthesize current evidence on the impact of iron metabolism on cardiac autonomic control, with a focus on potential mechanisms and clinical implications of this underappreciated complication.

## 2. Materials and Methods

This narrative review was conducted to provide a comprehensive and integrative overview of the relationship between iron metabolism disorders and autonomic nervous system dysfunction. A structured literature search was performed using PubMed (MEDLINE), Scopus, and Wiley Online Library (Supplementary Table S1). The search strategy included combinations of the following keywords: autonomic nervous system dysfunction, iron metabolism disorder, iron overload, iron deficiency, hereditary hemochromatosis, beta-thalassemia, and sickle cell anemia.

Publications from 1983 through 2025 were considered in order to capture both foundational and contemporary studies addressing autonomic function in the context of iron metabolism disturbances. No earlier time restriction was imposed to minimize the risk of omitting seminal research in this field. Only articles published in English were included.

The selected databases were chosen to ensure broad coverage of biomedical and interdisciplinary literature. Scopus indexes a substantial proportion of journals included in Web of Science, thereby reducing the likelihood of omitting relevant publications. Given that the primary focus of this review was pathophysiological and clinical associations rather than pharmacotherapy, omission of Embase was not considered likely to substantially affect the overall completeness of the literature identified.

Study selection was guided by thematic relevance rather than by a predefined PICO framework. Peer-reviewed original studies, clinical investigations, selected review articles, and mechanistic experimental studies relevant to autonomic dysfunction in iron deficiency or iron overload were considered eligible. Animal studies were included when they provided mechanistic insights with translational relevance to human physiology. Publications were excluded if they did not address autonomic outcomes or were unavailable in full text.

Titles and abstracts were screened to identify potentially relevant studies, followed by full-text evaluation. Extracted information included study design, population characteristics, type of iron metabolism disorder, autonomic assessment methods (e.g., heart rate variability, baroreflex sensitivity, ECG-derived markers), and principal findings. Due to heterogeneity in study designs, populations, and outcome measures, findings were synthesized qualitatively.

In total, 107 publications were included, comprising original research articles, review papers, and brief communications, with particular emphasis on cardiological manifestations of autonomic dysfunction.

## 3. Autonomic Regulation of Myocardial Function

The autonomic nervous system (ANS) comprises sympathetic and parasympathetic branches, each innervating target organs through distinct neural pathways and exerting largely antagonistic effects to maintain physiological homeostasis [10]. The sympathetic branch increases heart rate, contractility, and vascular tone, whereas the parasympathetic branch, primarily mediated by the vagus nerve, reduces heart rate. Within the cardiovascular system, sympathetic activation supports perfusion under stress but predisposes to arrhythmias, including ventricular tachyarrhythmias, through enhanced automaticity and triggered activity. In contrast, parasympathetic predominance stabilizes cardiac rhythm and confers protection against tachyarrhythmias; however, excessive vagal tone may promote bradyarrhythmias.

The ANS dynamically regulates cardiovascular, respiratory, and vascular function in response to internal and external demands. Disruption of this regulatory network, collectively termed dysautonomia, encompasses a heterogeneous group of conditions with diverse etiologies and clinical presentations [11]. These manifestations range from transient, neurally mediated hypotension to progressive neurodegenerative disorders. In certain conditions, such as pure autonomic failure and multiple system atrophy, autonomic dysfunction represents a primary pathological process, whereas in others it occurs secondary to, or exacerbates, underlying diseases including HF or obstructive sleep apnea [11]. The mechanisms underlying dysautonomia remain incompletely characterized, contributing to variability in clinical presentation and diagnostic uncertainty.

**Figure 1:**
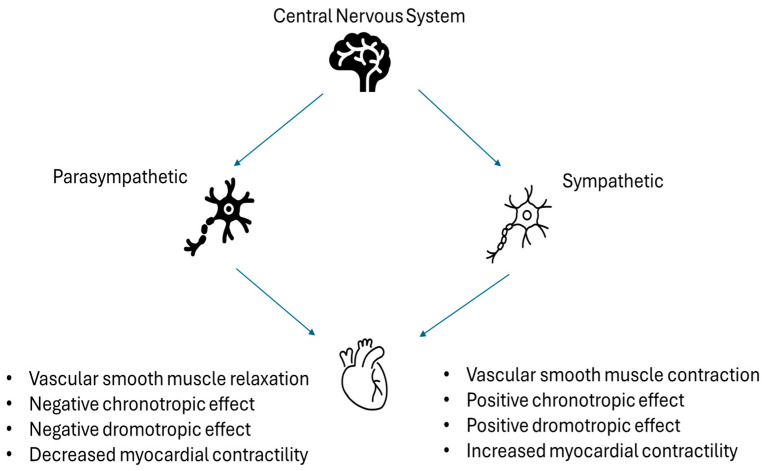
Autonomic nervous system and its two main divisions—the sympathetic and parasympathetic nervous systems—and their effects.

Recent studies have suggested a potential association between disturbances in iron metabolism and autonomic dysfunction [12,13,14,15]. Alterations in iron homeostasis have been implicated in impaired autonomic regulation across a range of clinical contexts; however, the strength, direction, and mechanistic basis of these associations remain uncertain. Accordingly, this systematic review therefore aims to synthesize the available evidence examining the relationship between iron metabolism and autonomic nervous system function (Figure 1).

### 3.1. Selected Methods for Assessing the Autonomic Nervous System Function

Several methods are used to assess the function of the ANS. From many techniques, one can distinguish the following: global and integrated tests (such as heart rate variability or BRS), cardiovascular autonomic reflex tests (deep breathing test, Valsalva maneuvers, 30:15 ratio, sustained handgrip test and cold pressor test) and electrophysiological indices of cardiac autonomic regulation (P-wave dispersion, QT-dispersion and ventricular late potentials) [16,17,18,19,20,21,22,23]. Together, these tests provide a comprehensive evaluation of autonomic regulation, helping to identify dysfunction in various neurological and systemic disorders.

#### 3.1.1. Heart Rate Variability

Heart rate variability (HRV), defined as beat-to-beat heart variation in heart period, is a fundamental measure for assessing ANS function [19,23]. HRV quantifies physiological fluctuations in the time intervals between consecutive heartbeats, reflecting the dynamic balance between sympathetic and parasympathetic modulation of the sinoatrial node [24]. Spectral analysis of HRV decomposes these fluctuations into high-frequency (HF), low-frequency (LF), and very-low-frequency (VLF) bands. HF power predominantly reflects parasympathetic activity, while LF power represents a composite of sympathetic and parasympathetic modulation [24]. Time-domain indices, such as root mean square of successive differences (RMSSD) primarily reflects parasympathetic modulation within the respiratory frequency range [25], while the HRV-triangular index has been shown to serves as an independent predictor of arrhythmic events and cardiac mortality after myocardial infarction [26].

Numerous studies have demonstrated that pathological conditions—including systemic inflammation, infection, or cardiovascular disease—are associated with reduced HRV, whereas higher HRV is characteristic of healthy individuals [27,28,29,30]. Clinically, diminished HRV has been identified as a prognostic marker for increased mortality following myocardial infarction, chronic heart failure (CHF), and diabetic neuropathy, and it correlates with fatigue severity in chronic fatigue syndrome [21,31]. Furthermore, reduced HRV has been linked to coronary artery disease and sudden cardiac death [32], chronic pain disorders [33], metabolic syndrome [34], depression [35], and bipolar disorder [36]. The Autonomic Tone and Reflexes After Myocardial Infarction (ATRAMI) study demonstrated that patients with low HRV and impaired BRS post-myocardial-infarction experienced worse clinical outcomes [21], a finding subsequently confirmed by later investigations [37,38]. Similarly, in patients with non-ischemic dilated cardiomyopathy, reduced HRV has been shown to predict cardiac mortality, underscoring its prognostic value in HF populations [39,40,41]. Moreover, frequency-domain HRV analysis has demonstrated strong predictive power for sudden cardiac death, suggesting that spectral indices may enhance risk stratification beyond traditional time-domain indices [42,43].

A multitude of physiological, pathological, psychological, environmental, and genetic factors influence HRV, with genetic polymorphisms modulating individual autonomic responses under stress [44].

#### 3.1.2. Baroreflex Sensitivity

Baroreflex sensitivity (BRS) quantifies the effectiveness with which arterial baroreceptors translate changes in blood pressure into reflexive adjustments of heart rate, thereby reflecting the integrity of autonomic cardiovascular control. Numerous studies have established that impaired BRS is strongly associated with an increased risk of ventricular fibrillation and cardiovascular mortality [18,21,22]. In a seminal canine model, Schwartz and colleagues demonstrated that animals with reduced BRS following myocardial infarction were markedly more susceptible to exercise-induced ventricular fibrillation, revealing an inverse relationship between BRS magnitude and arrhythmic vulnerability [18].

Translating these findings to humans, the ATRAMI study enrolled nearly 1300 post-infarction patients and identified a parasympathetic deficit—defined as BRS below 3 ms/mmHg—as an independent predictor of cardiovascular mortality, even after adjustment for conventional risk factors. Moreover, the combination of reduced left ventricular ejection fraction (LVEF) and low BRS substantially improved prognostic accuracy, with the highest mortality risk observed in patients under 65 years of age [21]. Subsequent investigations extended these findings to CHF populations, in which impaired BRS similarly predicted adverse clinical outcomes.

Mortara et al. assessed BRS using the phenylephrine method in 282 patients with HF and demonstrated that reduced BRS was an independent predictor of cardiac death or urgent transplantation after controlling for New York Hear Association (NYHA) class, LVEF, baseline R–R interval, and peak oxygen consumption [45,46]. Using a frequency-domain transfer-function technique, Pinna et al. further demonstrated that reduced oscillatory baroreflex gain in 317 clinically stable HF patients was associated with worse prognosis across NYHA classes, reinforcing the prognostic value of BRS as a marker of autonomic dysfunction and cardiovascular risk [47].

### 3.2. Integration of Autonomic Reflex Tests in the Comprehensive Assessment of Autonomic Nervous System Function

Cardiovascular autonomic reflex tests represent standardized, non-invasive procedures used to assess both parasympathetic and sympathetic components of autonomic cardiovascular regulation [17]. These tests evaluate the integrity of reflex pathways that modulate heart rate (HR) and blood pressure (BP) in response to defined physiological challenges. The deep breathing test measures respiratory sinus arrhythmia by analyzing HRV during controlled breathing; reduced variability indicates impaired parasympathetic function [17,48]. The Valsalva maneuver evaluates baroreflex-mediated adjustments in HR and BP across phases of increased intrathoracic pressure, providing information on both sympathetic and parasympathetic integrity [16,43].

The 30:15 Ratio (orthostatic test) evaluates the HR response upon standing—specifically, the ratio of the 30th to the 15th R–R interval after rising from a supine position—with a blunted ratio indicating vagal dysfunction [17]. The sustained handgrip test examines sympathetic vasoconstrictor function by measuring the rise in diastolic BP during isometric contraction with a subnormal increase indicating impaired sympathetic efferent activity [17,20]. Finally, the cold pressor test induces sympathetic activation through hand immersion in cold water, with inadequate BP response signifying sympathetic dysfunction [49].

Collectively, these reflex tests provide a comprehensive evaluation of autonomic control over cardiovascular function and are widely used for diagnosing of conditions such as diabetic autonomic neuropathy, dysautonomia, and other disorders affecting autonomic regulation [17].

### 3.3. Electrophysiological Tests

Electrophysiological indices of cardiac autonomic regulation provide useful insights into the potential influence of sympathetic and parasympathetic activity on cardiac electrical stability and conduction [50,51,52,53]. These measures, derived from detailed electrocardiographic (ECG) analyses, can reflect subtle alterations in myocardial excitability and repolarization dynamics. P-wave dispersion (PWD), defined as the difference between the longest and shortest P-wave durations across ECG leads, is considered a marker of atrial conduction heterogeneity and parasympathetic modulation. Increased PWD may indicate a higher susceptibility to atrial arrhythmias, potentially reflecting autonomic imbalance or structural atrial remodeling [51,54,55,56].

QT dispersion (QTd), which represents variability in QT intervals across ECG leads, reflects spatial inhomogeneity of ventricular repolarization. Elevated QTd may indicate increased electrical instability and is thought to be influenced by sympathetic tone, making it a potential predictor of ventricular arrhythmias and sudden cardiac death [57,58,59]. Ventricular late potentials (VLPs), detected using signal-averaged ECG, correspond to low-amplitude, high-frequency signals occurring at the terminal portion of the QRS complex. These signals may reflect delayed myocardial depolarization in regions of slow conduction, which could act as substrates for re-entrant ventricular arrhythmias. The presence of VLPs has been associated with sympathetic overactivity and structural myocardial damage, particularly following myocardial infarction [60,61].

Overall, these electrophysiological parameters serve as non-invasive markers that may indicate autonomic imbalance and myocardial electrical vulnerability, potentially contributing to risk stratification in patients with ischemic heart disease, HF, and other arrhythmogenic conditions.

## 4. Autonomic Dysfunction in Iron Metabolism Disorders: Insights from the Literature

Autonomic dysfunction in iron-metabolism disorders may result from an imbalance between sympathetic and parasympathetic activity, often characterized by a shift toward sympathetic predominance. Consistent with this notion, abnormalities in HRV and BRS have been observed in patients with iron-related disorders [62,63], suggesting that iron homeostasis may play important role in maintaining ANS integrity. In the context of chronic ID, sustained tissue hypoxia may reduce the chemosensory responsiveness of the carotid body, potentially impairing autonomic reflex control [12].

While the pathophysiological relationships between autonomic dysfunction and conditions such as hypertension, HF, and diabetes are well established [64,65,66,67,68], evidence linking iron-metabolism disorders to autonomic abnormalities remains relatively limited. Both IO and ID can contribute to systemic complications through distinct biological mechanisms [69,70,71]; however, emerging data from small-cohort studies suggest that ANS impairment may also occur in these disorders [9,72,73,74].

In ID, autonomic dysfunction are thought to arise primarily from hypoxia-driven effects on autonomic control. Prolonged oxygen deprivation may attenuate carotid body sensitivity, leading to blunted reflexive autonomic responses and altered cardiovascular regulation [12]. Early evidence of ANS impairment in ID was reported by Nand et al. (1989), who evaluated 30 individuals with chronic anemia using deep breathing, Valsalva, cold-pressor, and atropine challenge tests; all participants demonstrated at least one abnormal response compared with controls, indicating widespread autonomic irregularities [75]. Subsequent studies have reinforced these findings. For example, Yokusoglu et al. reported reduced parasympathetic tone and overall autonomic imbalance in ID anemia patients using HRV analysis [12], while Jibhkate et al. observed that 78% of 60 ID anemia subjects exhibited abnormalities in at least one autonomic reflex test, suggesting receptor-level dysfunction and sympathetic predominance [14].

Within the broader spectrum of ID disorders, only a few studies have systematically examined HRV-based indices. In a cohort of 43 ID anemia patients, Yokusoglu et al. found reductions in most time-domain HRV indices, with the exception of RMSSD and the HRV-triangular index compared with healthy controls [12]. These alterations have been interpreted as indicative of increased sympathetic activity in ID individuals [12,25,26]. By contrast, Tuncer et al. did not observe these HRV findings in a smaller group of hospitalized ID anemia patients, likely due to methodological differences and limited sample size [62]. Continuous, ambulatory recordings—as used by Yokusoglu et al.—may more accurately capture hypoxia-driven fluctuations in autonomic activity than short, in-hospital measurements [12].

Collectively, these studies suggest that hypoxia associated ID may contribute to autonomic imbalance, with reduced parasympathetic modulation and relatively increased sympathetic drive. Although the precise molecular and neurophysiological mechanisms remain to be fully elucidated, the available evidence highlights a potential link between disordered iron metabolism and altered autonomic regulation, warranting further targeted investigation.

Autonomic dysfunction in iron-metabolism disorders may result from an imbalance between sympathetic and parasympathetic activity, often characterized by a relative shift toward sympathetic predominance. Consistent with this possibility, abnormalities in HRV and BRS have been observed in patients with iron-related disorders [62,63], suggesting that iron homeostasis may play an important role in maintaining ANS integrity. In chronic ID sustained tissue hypoxia may reduce to chemosensory responsiveness of carotid body, potentially affecting autonomic reflex control [12].

Although the pathophysiological relationships between autonomic dysfunction and conditions such as hypertension, HF, and diabetes are well established [64,65,66,67,68], evidence linking iron-metabolism disorders to similar autonomic abnormalities remains limited. Both IO and ID contribute to systemic complications through distinct biological mechanisms [69,70,71]; however emerging data from small-cohort studies indicate that ANS impairment may also occur in these conditions [9,72,73,74].

While the precise molecular and neurophysiological mechanisms underlying autonomic dysfunction in iron disorders remain to be fully elucidated, the existing evidence highlights a potential link between disordered iron metabolism and altered autonomic regulation, representing an area that warrants further targeted investigation.

### 4.1. Autonomic Nervous System Alterations in Iron Overload

Individuals with IO exhibit elevated levels of non–transferrin-bound iron, which promotes ROS formation and may influence central sympathetic regulatory centers, potentially contributing to sympathetic predominance [76]. ROS including nitric oxide (NO), superoxide (O_2_^−^), and peroxynitrite (ONOO^−^)-participate in oxidative-stress–dependent reactions within the cardiovascular microenvironment and can modulate cardiac autonomic signaling [76,77]. Under physiological conditions, ROS can exert a stimulatory influence on sympathetic outflow, whereas NO provides a counter-regulatory inhibitory influence, together supporting balanced autonomic control of heart rate and vascular tone [78].

In IO states, saturation of transferrin-binding capacity leads to NTBI accumulation. NTBI’s high pro-oxidant activity promotes Haber–Weiss and Fenton reactions, increasing ROS production [76]. Elevated ROS levels within central autonomic nuclei—particularly the rostral ventrolateral medulla (RVLM), a pivotal vasomotor center—may enhance sympathetic regulatory activity., Angiotensin II acting through AT_1_ receptors can further amplify mitochondrial ROS generation in the RVLM and activate caspase-3–dependent signaling via the Ras/p38 MAPK cascade, potentially contributing sympathetic overactivity [8,76].

Experimental studies support these mechanisms. Kishi and colleagues reported that spontaneously hypertensive rats (SHR) and stroke-prone SHR (SHRSP) exhibited higher sympathetic activity and increased ROS concentrations in the RVLM compared with normotensive controls, accompanied by reduced expression of the ROS-scavenging enzyme superoxide dismutase [8]. Angiotensin II–induced AT_1_ stimulation also triggered caspase-3 activation, which, appeared to enhance sympathetic drive in SHRSP animals [8]. Additionally, Jumrussirikul et al. demonstrated that uncoupling of neuronal nitric oxide synthase (nNOS) in tetrahydrobiopterin (BH_4_)–deficient rats impaired heart-rate responses to vagal stimulation more than complete nNOS gene deletion, suggesting that ROS can interfere with NO-mediated parasympathetic signaling and diminish vagally mediated bradycardia [79].

**Figure 2:**
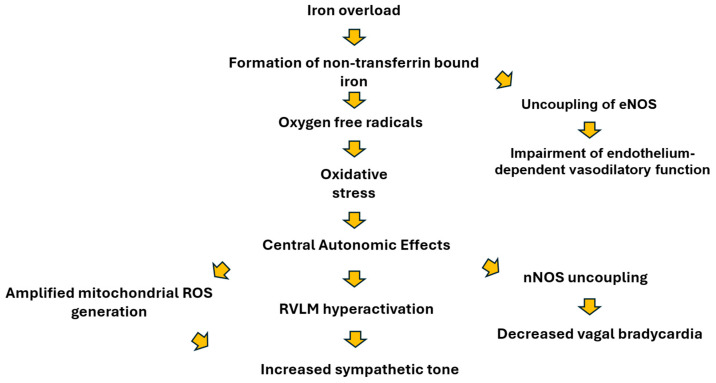
Potential mechanisms of iron-induced sympathetic overactivity in IO.

Clinical and experimental data further indicate that oxidative stress may affect autonomic regulation. In patients with HF, intravenous antioxidant administration improved autonomic responsiveness compared to healthy controls [80]. In a rat model of post-infarction HF, antioxidant treatment attenuated sympathetic activity, evidenced by smaller reductions in heart rate and mean arterial pressure during ganglionic blockade with hexamethonium and lower urinary norepinephrine excretion [81]. Complementary experimental work by Cardoso et al. showed that rats with elevated serum or tissue iron levels exhibited altered BRS assessed via phenylephrine and sodium nitroprusside; administration of the iron chelator deferoxamine restored baroreceptor function to control levels, suggesting that iron-related autonomic changes may be at least partially reversible [9] (Figure 2).

### 4.2. Beta-Thalassemia Major and Autonomic Nervous System Dysfunction: A Literature Review

Among IO disorders, autonomic dysfunction is most extensively studied in patients with beta-thalassemia major [74,82,83,84]. Beta-thalassemia results from inherited defects in β-globin synthesis and presents in three clinical forms—minor, intermedia, and major—with the major form being the most severe [85]. Symptoms of hemolytic anemia typically appear by six months of age, necessitating lifelong red blood cell transfusions and, frequently, iron-chelation therapy [86]. Despite chronic anemia, these patients develop IO due to both repeated transfusions and ineffective erythropoiesis [86].

Franzoni et al. evaluated 19 transfusion-dependent, cardiac-uncomplicated beta-thalassemia major patients using stress echocardiography and 24 h Holter monitoring [87]. Time- and frequency-domain HRV indices were reduced compared with healthy controls, and VLPs appeared in six patients, four of whom experienced non-sustained ventricular tachycardia. VLPs likely reflect heterogeneous depolarization secondary to myocardial iron deposition and fibrosis [88], while reduced HRV suggests impaired parasympathetic modulation in the context of chronic anemia [74]. Kardelen et al. confirmed these findings in 32 beta-thalassemia major patients, demonstrating consistently lower HRV parameters relative to controls [82].

Stamboulis et al. examined 39 patients using six standardized autonomic function tests—including tilt testing, hand-grip response, and sympathetic skin response for sympathetic function, as well as R–R interval variation, deep-breathing, and the 30/15 ratio for parasympathetic function [83]. Although overall autonomic dysfunction was more prevalent than in controls, the rates of isolated parasympathetic (5%) and sympathetic (10%) impairment were comparable between groups.

Salama et al. assessed 30 pediatric beta-thalassemia major patients with resting 12-lead ECG and transthoracic echocardiography, reporting prolonged PWD and QTd—markers of atrial conduction heterogeneity and ventricular repolarization inhomogeneity [84]. Both PWD and QTd correlated strongly with serum ferritin levels and echocardiographic evidence of myocardial involvement. PWD may indicate atrial remodeling and arrhythmia risk, while QTd distinguishes homogeneous from heterogeneous ventricular repolarization [89,90,91].

Taken together, these studies suggest that IO in beta-thalassemia major—potentially mediated by myocardial iron deposition, fibrosis, and oxidative stress—is associated with altered autonomic balance, characterized by reduced parasympathetic tone, relative sympathetic overactivity, and electrophysiological instability

### 4.3. Hereditary Hemochromatosis and Autonomic Nervous System Dysfunction: A Literature Review

IO is the hallmark laboratory abnormality in hereditary hemochromatosis (HH), one of the most prevalent autosomal recessive disorders in Caucasian populations, with an estimated incidence of approximately 1 in 200 individuals [3]. At least five pathogenic HFE gene mutations have been identified, each exhibiting variable penetrance [3]. Although the mechanisms linking HH to autonomic function remain incompletely characterized, emerging evidence suggests that iron accumulation may affect both sympathetic and parasympathetic branches of the ANS and that these effects may be at least partially reversible [15,92,93].

Seravalle et al. evaluated 18 male patients with newly diagnosed HH by measuring beat-to-beat blood pressure, heart rate (HR), electrocardiogram (ECG), and muscle sympathetic nerve activity (MSNA) before and after serial venesections [92]. Following venesection, high-frequency (HF) components of HRV increased, while low-frequency (LF) components of systolic blood-pressure variability decreased. Baseline MSNA correlated positively with transferrin saturation, hepatic iron concentration, and total iron removed; reductions in MSNA after venesections were associated with improvements in the baroreflex-MSNA index, reflecting spontaneous sympathetic modulation by diastolic pressure and cardiac interval [92]. These findings indicate that IO in HH is linked with increased sympathetic activity relative to iron burden, and that regular venesections may partially restore sympathetic tone, with transferrin saturation emerging as the strongest predictor of recovery [92,94].

Notably, patients also exhibited reduced HF HRV at baseline, suggesting parasympathetic impairment alongside sympathetic predominance. Both HF and LF abnormalities tended to normalize following venesections [95]. Resting HR, however, did not differ from controls, highlighting its limited sensitivity to subclinical autonomic disturbances [95].

### 4.4. Iron Deficiency–Related Diseases and Autonomic Dysfunction

The most direct evidence linking ID to autonomic dysfunction comes from studies among patients with ID anemia, which consistently report reduced HRV and blunted BRS, indicative of parasympathetic withdrawal and sympathetic predominance [12,72,73,74]. Beyond hematological manifestations, neurological conditions associated with central ID, such as restless legs syndrome (RLS), have been linked to heightened sympathetic activity, altered HRV, and nocturnal hypertension [96,97]. Similarly, patients with chronic fatigue syndrome (CFS) and reduced ferritin levels exhibit diminished HRV and postural tachycardia, suggesting that iron-dependent mitochondrial dysfunction may contribute to autonomic imbalance [31].

Patients with CHF are prone to ID because of depletion of iron stores (absolute ID) or, more commonly, as a result of impaired iron metabolism during inflammatory processes characteristic of CHF (functional ID). In CHF, pro-inflammatory cytokines are activated, which block intestinal iron absorption and redirect iron from the circulation to the reticuloendothelial system. As a result, impaired absorption of this element from the gastrointestinal tract and increased iron sequestration in cells leads to ID. It is estimated that approximately half of HF patients suffer from ID, with values ranging from 47 to 68% [98,99]. Overall, a slightly higher prevalence has been observed in HF with preserved ejection fraction (HFpEF) compared to HF with mildly reduced (HFmrEF) or reduced ejection fraction (HFrEF) [100]. Patients with more severe HF are at greater risk of ID and anemia. In patients with acute decompensated HF, ID was observed in 54% of patients with HFrEF and 56% of patients with HFpEF, but only in HFrEF was it independently associated with a longer hospital stay [101].

Importantly, in CHF, ID—independent of anemia—has been associated with sympathetic overactivity, impaired baroreflex function, and worse cardiovascular outcomes. Conversely, intravenous iron repletion has been shown to improve autonomic indices and exercise capacity [102,103]. Developmental ID has been linked to disrupted autonomic maturation, with infants and children exhibiting reduced HRV, altered sleep-related autonomic patterns, and long-term dysregulation of cardiac vagal tone [104,105,106,107,108]. Collectively, these findings suggests that both absolute and functional ID can lead to early and measurable disturbances in autonomic regulation, highlighting the importance of routine autonomic assessment in iron-deficient populations to identify subclinical dysfunction and potentially mitigate cardiovascular risk.

## 5. Summary of the Literature on Autonomic Dysfunction in Iron Disorders

As part of the summary, a comprehensive review was conducted of studies focusing on autonomic nervous system dysfunction among patients with iron metabolism disorders. Peer-reviewed articles published between 1983 and 2025 were included, selecting only those with adequately powered sample sizes. Reference lists of all eligible papers were hand-searched to identify additional relevant studies, yielding a total of 15 publications. These investigations spanned diverse geographic regions and encompassed study populations aged 5 months to 76 years. Key findings are synthesized in Table 1 and Table 2. Methodological Quality Assessment are summarized in Table 3.

### 5.1. Methodological Quality—Iron Deficiency

Among studies evaluating iron deficiency, the overall methodological quality was predominantly moderate. Most investigations were observational, single-center studies with limited sample sizes. Although control groups were generally included, formal adjustment for potential confounders—such as age, cardiovascular comorbidities, medication use, and metabolic parameters—was inconsistently reported. In pediatric cohorts, small sample size and limited reporting of selection methods contributed to a higher estimated risk of bias in several studies. Overall, the strength of evidence for ID-related autonomic dysfunction is supported by consistent findings across studies but remains limited by methodological heterogeneity and incomplete confounder control.

### 5.2. Methodological Quality—Iron Overload

Studies investigating iron overload also demonstrated predominantly moderate methodological quality. Most were case–control or cross-sectional in design, with relatively small to moderate sample sizes. While autonomic assessment methods were generally well described (e.g., HRV, baroreflex sensitivity, MSNA), adjustment for confounding variables was frequently incomplete, particularly regarding cardiovascular risk factors and treatment status (e.g., chelation therapy). Some studies lacked healthy control groups, limiting causal inference. The available evidence consistently suggests autonomic alterations in iron overload states; however, the overall level of evidence is constrained by study design and risk of residual confounding.

## 6. Conclusions

Iron metabolism disorders are among the most prevalent micronutrient imbalances worldwide, and, while overt autonomic symptoms are often absent, subtle alterations in autonomic function have been reported in affected populations. Available human data suggest associations between iron imbalance and autonomic dysregulation, with mechanistic links—such as iron-related oxidative stress, gap-junction dysfunction, and impaired nitric oxide signaling—largely inferred from experimental and preclinical models rather than directly demonstrated in clinical studies. Observational evidence further indicates that correction of iron homeostasis, either through chelation in overload states or iron repletion in deficiency, may be accompanied by partial normalization of autonomic indices; however, these findings remain associative and cannot establish causality. Consequently, the potential reversibility of autonomic disturbances should be regarded as hypothesis-generating rather than definitive. Although autonomic assessment methods were generally well described and consistent patterns of autonomic imbalance were observed, adjustment for key confounding variables—such as age, cardiovascular comorbidities, medication use, metabolic status, and treatment exposure—was inconsistently reported. In ID cohorts, particularly pediatric populations, limited sample size and incomplete characterization of baseline cardiovascular risk may have influenced the magnitude of reported autonomic alterations. Similarly, in iron overload states, heterogeneity in underlying conditions (e.g., hereditary hemochromatosis versus transfusion-related iron overload) and treatment status may contribute to variability in autonomic outcomes. Therefore, while the overall direction of evidence supports an association between iron status and autonomic dysfunction, causality cannot be firmly established, and residual confounding cannot be excluded. Future studies with larger, multicenter cohorts and standardized autonomic assessment protocols are warranted to strengthen the level of evidence. These considerations highlight the need for well-designed, prospective, and adequately controlled multicenter studies employing standardized autonomic testing to clarify the prognostic relevance of autonomic tone alterations in iron-disordered populations. Insufficient adjustment for metabolic and cardiovascular confounders in existing studies limits the ability to attribute observed autonomic changes specifically to iron status.

## Figures and Tables

**Table 1:** (**A**) Iron Deficiency—Human Studies (Adult). (**B**) Iron Deficiency—Human Studies (Pediatric/Young). (**C**) Mixed/Secondary Iron-Related Conditions.

Author, Year	Population	Condition	ANS Method	Main Autonomic Finding	Clinical vs. Surrogate	Confounder Control	Estimated Quality
(**A**)							
Yokusoglu 2007 [12]	Adults	Iron Deficiency Anemia	24 h HRV (time-domain)	↓ SDNN, ↓ SDANN, ↓ pNN50	Surrogate	Not reported	Moderate
Martins 2012 [106] (IDA subgroup)	Adults	Iron Deficiency Anemia	HRV, baroreflex, autonomic tests	Reduced parasympathetic modulation	Surrogate	Not reported	Moderate
(**B**)							
Medigue 1996 [74]	Pediatric (6–7 months)	Iron Deficiency Anemia	Polysomnography + ECG	Delayed vagal maturation	Surrogate	Not reported	Low–Moderate
Kolkiran 2005 [105]	Pediatric (2–22 years)	IDA with breath-holding spells	ECG during episodes	↓ Respiratory sinus arrhythmia	Surrogate	Not reported	Moderate
Jibhkate 2019 [14]	Adults (20–40 years)	Iron Deficiency Anemia	Autonomic reflex tests	78% showed autonomic dysfunction	Clinical + Surrogate	Not reported	Moderate
(**C**)							
Ando 1996 [73]	Adults	FAP + anemia	Symptom score	Anemia severity correlated with autonomic dysfunction	Clinical	Not reported	Low
Martins 2012 [106] (SCA/SCT)	Adults	Sickle Cell Disease	HRV, BRS, reflex tests	↓ BRS, ↓ parasympathetic modulation	Surrogate	Not reported	Moderate

↓ decrease.

**Table 2:** (**A**) Iron Overload—Human Studies (Adults). (**B**) Iron Overload—Humans Studies (Pediatric/Young). (**C**) Iron Overload—Animal Studies.

Author, Year	Population	Condition	ANS Method	Main Autonomic Finding	Clinical vs. Surrogate	Confounder Control	Estimated Quality
(**A**)							
Seravalle 2016 [108]	Adults	Hereditary Hemochromatosis	MSNA, HRV, baroreflex	↑ MSNA, reversible after iron depletion	Surrogate	Partial	Moderate
Stamboulis 2012 [83]	Adults	Beta-thalassemia	Autonomic reflex tests	Higher prevalence of ANS dysfunction	Surrogate	Not reported	Moderate
(**B**)							
Franzoni 2004 [87]	Pediatric/Young	Thalassemia Major	24 h HRV + VLP	↓ HRV, ↑ arrhythmias	Clinical + Surrogate	Not reported	Moderate
Kardelen 2008 [82]	Pediatric	Thalassemia Major	24 h HRV (time + frequency)	All HRV parameters reduced	Surrogate	Not reported	Moderate
Yuksel 2016 [109]	Young Adults	Beta-thalassemia Major	HRR, MRI T2*	HRR correlated with iron load	Surrogate	Not reported	Moderate
Salama 2020 [84]	Pediatric	Transfusion-dependent β-TM	ECG (PWd, QT)	Prolonged PWd and QT dispersion	Clinical + Surrogate	Not reported	Moderate
Wijarnpreecha 2015 [107]	Young Adults	Transfusion-independent thalassemia	HRV + MRI T2*	HRV correlated with NTBI and ferritin	Surrogate	No control group	Low–Moderate
(**C**)							
Cardoso 2005 [9]	Animal (Rat model)	Tissue Iron Overload	Baroreflex sensitivity	↑ Baroreceptor gain (reversible with deferoxamine)	Mechanistic	Not applicable	Moderate

↑ increase, ↓ decrease.

**Table 3:** Methodological Quality Assessment (Newcastle-Ottawa Scale Adaptation).

Study	Design	Selection (0–4)	Comparability (0–2)	Outcome/Exposure (0–3)	Total Score (0–9)	Overall Risk of Bias
Yokusoglu 2007 [12]	Case–control	3	1	2	6	Moderate
Martins 2012 [106]	Case–control	3	1	2	6	Moderate
Medigue 1996 [74]	Case–control	2	0	2	4	High
Kolkiran 2005 [105]	Case–control	3	0	2	5	Moderate
Jibhkate 2019 [14]	Cross–sectional	3	0	2	5	Moderate
Ando 1996 [73]	Observational	2	0	1	3	High
Stamboulis 2012 [83]	Case–control	3	1	2	6	Moderate
Franzoni 2004 [87]	Case–control	3	0	2	5	Moderate
Kardelen 2008 [82]	Case–control	3	0	2	5	Moderate
Wijarnpreecha 2015 [107]	Cross-sectional (no control)	2	0	2	4	High
Seravalle 2016 [108]	Interventional (before-after)	3	1	2	6	Moderate
Yuksel 2016 [109]	Cross-sectional	3	0	2	5	Moderate
Salama 2020 [84]	Case–control	3	0	2	5	Moderate
Cardoso 2005 [9]	Animal experimental	N/A	N/A	N/A	N/A	Not applicable

Note: The Newcastle–Ottawa Scale (NOS) was adapted for observational studies. Animal experimental studies were not assessed using NOS due to methodological differences.

## Data Availability

Data are available from the corresponding author upon reasonable request.

## Supplementary Materials

The following supporting information can be downloaded at https://www.mdpi.com/article/10.3390/jcm15051871/s1, Table S1: Full Search Strategies for All Databases.

## References

[1] Chompoopong P., Mauermann M.L., Siddiqi H., Peltier A. (2024). Amyloid Neuropathy: From Pathophysiology to Treatment in Light-Chain Amyloidosis and Hereditary Transthyretin Amyloidosis. Ann. Neurol..

[2] Briguglio M., Hrelia S., Malaguti M., Lombardi G., Riso P., Porrini M., Perazzo P., Banfi G. (2020). The Central Role of Iron in Human Nutrition: From Folk to Contemporary Medicine. Nutrients.

[3] Daniłowicz-Szymanowicz L., Świątczak M., Sikorska K., Starzyński R.R., Raczak A., Lipiński P. (2021). Pathogenesis, Diagnosis, and Clinical Implications of Hereditary Hemochromatosis-The Cardiological Point of View. Diagnostics.

[4] Ungvari Z., Kaley G., de Cabo R., Sonntag W.E., Csiszar A. (2010). Mechanisms of vascular aging: New perspectives. J. Gerontol. A Biol. Sci. Med. Sci..

[5] Andrès E., Lorenzo-Villalba N. (2025). Carence fonctionnelle en fer: Une entité controversée, un défi clinique. Cah. Santé Médecine Thérapeutique.

[6] Duca L., Di Pierro E., Scaramellini N., Granata F., Graziadei G. (2025). The Relationship Between Non-Transferrin-Bound Iron (NTBI), Labile Plasma Iron (LPI), and Iron Toxicity. Int. J. Mol. Sci..

[7] Danson E.J., Paterson D.J. (2006). Reactive oxygen species and autonomic regulation of cardiac excitability. J. Cardiovasc. Electrophysiol..

[8] Kishi T., Hirooka Y., Kimura Y., Ito K., Shimokawa H., Takeshita A. (2004). Increased reactive oxygen species in rostral ventrolateral medulla contribute to neural mechanisms of hypertension in stroke-prone spontaneously hypertensive rats. Circulation.

[9] Cardoso L.M., Pedrosa M.L., Silva M.E., Moraes M., Colombari E., Chianca D. (2005). Baroreflex function in conscious rats submitted to iron overload. Braz. J. Med. Biol. Res..

[10] McCorry L.K. (2007). Physiology of the autonomic nervous system. Am. J. Pharm. Educ..

[11] McLeod K.A. (2001). Dysautonomia and neurocardiogenic syncope. Curr. Opin. Cardiol..

[12] Yokusoglu M., Nevruz O., Baysan O., Uzun M., Demirkol S., Avcu F., Koz C., Çetin T., Hasimi A., Ural A.U. (2007). The altered autonomic nervous system activity in iron deficiency anemia. Tohoku J. Exp. Med..

[13] Garringer H.J., Irimia J.M., Li W., Goodwin C.B., Richine B., Acton A., Chan R.J., Peacock M., Muhoberac B.B., Ghetti B. (2016). Effect of Systemic Iron Overload and a Chelation Therapy in a Mouse Model of the Neurodegenerative Disease Hereditary Ferritinopathy. PLoS ONE.

[14] Jibhkate A.N., Lath R.K. (2019). Assessing severity of involvement of autonomic functions in iron-deficiency anemia patients. Natl. J. Physiol. Pharm. Pharmacol..

[15] Sumneang N., Siri-Angkul N., Kumfu S., Chattipakorn S.C., Chattipakorn N. (2020). The effects of iron overload on mitochondrial function, mitochondrial dynamics, and ferroptosis in cardiomyocytes. Arch. Biochem. Biophys..

[16] Trimarco B., Volpe M., Ricciardelli B., Vigorito C., De Luca N., SaccÀ L., Condorelli M. (1983). Valsalva maneuver in the assessment of baroreflex responsiveness in borderline hypertensives. Cardiology.

[17] Ewing D.J., Martyn C.N., Young R.J., Clarke B.F. (1985). The value of cardiovascular autonomic function tests: 10 years experience in diabetes. Diabetes Care.

[18] Schwartz P.J., Vanoli E., Stramba-Badiale M., De Ferrari G.M., E Billman G., Foreman R.D. (1988). Autonomic mechanisms and sudden death. New insights from analysis of baroreceptor reflexes in conscious dogs with and without a myocardial infarction. Circulation.

[19] Omboni S., Parati G., Di Rienzo M., Wieling W., Mancia G. (1996). Blood pressure and heart rate variability in autonomic disorders: A critical review. Clin. Auton. Res..

[20] Khurana R.K., Setty A. (1996). The value of the isometric hand-grip test—Studies in various autonomic disorders. Clin. Auton. Res..

[21] La Rovere M.T., Bigger J.T., Marcus F.I., Mortara A., Schwartz P.J. (1998). Baroreflex sensitivity and heart-rate variability in prediction of total cardiac mortality after myocardial infarction. ATRAMI (Autonomic Tone and Reflexes After Myocardial Infarction) Investigators. Lancet.

[22] Swenne C.A. (2013). Baroreflex sensitivity: Mechanisms and measurement. Neth. Heart J..

[23] Shaffer F., McCraty R., Zerr C.L. (2014). A healthy heart is not a metronome: An integrative review of the heart’s anatomy and heart rate variability. Front. Psychol..

[24] Cygankiewicz I., Zareba W. (2013). Heart rate variability. Handb. Clin. Neurol..

[25] Galluzzi S., Nicosia F., Geroldi C., Alicandri A., Bonetti M., Romanelli G., Zulli R., Frisoni G.B. (2009). Cardiac autonomic dysfunction is associated with white matter lesions in patients with mild cognitive impairment. J. Gerontol. A Biol. Sci. Med. Sci..

[26] Frenneaux M.P. (2004). Autonomic changes in patients with heart failure and in post-myocardial infarction patients. Heart.

[27] Stein P.K., Bosner M.S., Kleiger R.E., Conger B.M. (1994). Heart rate variability: A measure of cardiac autonomic tone. Am. Heart J..

[28] Sztajzel J. (2004). Heart rate variability: A noninvasive electrocardiographic method to measure the autonomic nervous system. Swiss Med. Wkly..

[29] Thayer J.F., Yamamoto S.S., Brosschot J.F. (2010). The relationship of autonomic imbalance, heart rate variability and cardiovascular disease risk factors. Int. J. Cardiol..

[30] Hayano J., Yuda E. (2019). Pitfalls of assessment of autonomic function by heart rate variability. J. Physiol. Anthropol..

[31] Meeus M., Goubert D., De Backer F., Struyf F., Hermans L., Coppieters I., De Wandele I., Da Silva H., Calders P. (2013). Heart rate variability in patients with fibromyalgia and patients with chronic fatigue syndrome: A systematic review. Semin. Arthritis Rheum..

[32] Xhyheri B., Manfrini O., Mazzolini M., Pizzi C., Bugiardini R. (2012). Heart rate variability today. Prog. Cardiovasc. Dis..

[33] Broucqsault-Dédrie C., De Jonckheere J., Jeanne M., Nseir S. (2016). Measurement of Heart Rate Variability to Assess Pain in Sedated Critically Ill Patients: A Prospective Observational Study. PLoS ONE.

[34] Stuckey M.I., Tulppo M.P., Kiviniemi A.M., Petrella R.J. (2014). Heart rate variability and the metabolic syndrome: A systematic review of the literature. Diabetes Metab. Res. Rev..

[35] Koenig J., Kemp A.H., Beauchaine T.P., Thayer J.F., Kaess M. (2016). Depression and resting state heart rate variability in children and adolescents—A systematic review and meta-analysis. Clin. Psychol. Rev..

[36] Bassett D. (2016). A literature review of heart rate variability in depressive and bipolar disorders. Aust. N. Z. J. Psychiatry.

[37] Vybiral T., Glaeser D.H., Morris G., Hess K.R., Yang K., Francis M., Pratt C.M. (1993). Effects of low dose transdermal scopolamine on heart rate variability in acute myocardial infarction. J. Am. Coll. Cardiol..

[38] Taralov Z.Z., Terziyski K.V., Kostianev S.S. (2015). Heart Rate Variability as a Method for Assessment of the Autonomic Nervous System and the Adaptations to Different Physiological and Pathological Conditions. Folia Med..

[39] Fauchier L., Babuty D., Cosnay P., Autret M.L., Fauchier J.P. (1997). Heart rate variability in idiopathic dilated cardiomyopathy: Characteristics and prognostic value. J. Am. Coll. Cardiol..

[40] Fauchier L., Babuty D., Cosnay P., Fauchier J.P. (1999). Prognostic value of heart rate variability for sudden death and major arrhythmic events in patients with idiopathic dilated cardiomyopathy. J. Am. Coll. Cardiol..

[41] Karcz M., Chojnowska L., Zareba W., Ruzyłło W. (2003). Prognostic significance of heart rate variability in dilated cardiomyopathy. Int. J. Cardiol..

[42] Kiviniemi A.M., Tulppo M.P., Wichterle D., Hautala A.J., Tiinanen S., Seppänen T., Mäkikallio T.H., Huikuri H.V. (2007). Novel spectral indexes of heart rate variability as predictors of sudden and non-sudden cardiac death after an acute myocardial infarction. Ann. Med..

[43] Valkama J.O., Huikuri H.V., Airaksinen K.E., Linnaluoto M., Takkunen J. (1993). Changes in frequency domain measures of heart rate variability in relation to the onset of ventricular tachycardia in acute myocardial infarction. Int. J. Cardiol..

[44] Golosheykin S., Grant J.D., Novak O.V., Heath A.C., Anokhin A.P. (2017). Genetic influences on heart rate variability. Int. J. Psychophysiol..

[45] Mortara A., La Rovere M.T., Pinna G.D., Prpa A., Maestri R., Febo O., Pozzoli M., Opasich C., Tavazzi L. (1997). Arterial baroreflex modulation of heart rate in chronic heart failure: Clinical and hemodynamic correlates and prognostic implications. Circulation.

[46] Kaufmann D.K., Raczak G., Szwoch M., Wabich E., Świątczak M., Daniłowicz-Szymanowicz L. (2022). Baroreflex sensitivity but not microvolt T-wave alternans can predict major adverse cardiac events in ischemic heart failure. Cardiol. J..

[47] Pinna G.D., Maestri R., Capomolla S., Febo O., Robbi E., Cobelli F., La Rovere M.T. (2005). Applicability and clinical relevance of the transfer function method in the assessment of baroreflex sensitivity in heart failure patients. J. Am. Coll. Cardiol..

[48] Araujo C.G., Nobrega A.C., Castro C.L. (1992). Heart rate responses to deep breathing and 4-seconds of exercise before and after pharmacological blockade with atropine and propranolol. Clin. Auton. Res..

[49] Lamotte G., Boes C.J., Low P.A., Coon E.A. (2021). The expanding role of the cold pressor test: A brief history. Clin. Auton. Res..

[50] Breithardt G., Borggrefe M. (1987). Recent advances in the identification of patients at risk of ventricular tachyarrhythmias: Role of ventricular late potentials. Circulation.

[51] Dilaveris P.E., Gialafos J.E. (2001). P-wave dispersion: A novel predictor of paroxysmal atrial fibrillation. Ann. Noninvasive Electrocardiol..

[52] Franciosi S., Perry F.K.G., Roston T.M., Armstrong K.R., Claydon V.E., Sanatani S. (2017). The role of the autonomic nervous system in arrhythmias and sudden cardiac death. Auton. Neurosci..

[53] Kim H.G., Cheon E.J., Bai D.S., Lee Y.H., Koo B.-H. (2018). Stress and Heart Rate Variability: A Meta-Analysis and Review of the Literature. Psychiatry Investig..

[54] Aytemir K., Ozer N., Atalar E., Sade E., Aksöyek S., Övünç K., Oto A., Özmen F., Kes S. (2000). P wave dispersion on 12-lead electrocardiography in patients with paroxysmal atrial fibrillation. Pacing Clin. Electrophysiol..

[55] Yazici M., Ozdemir K., Altunkeser B.B., Kayrak M., Duzenli M.A., Vatankulu M.A., Soylu A., Ulgen M.S. (2007). The effect of diabetes mellitus on the P-wave dispersion. Circ. J..

[56] Yılmaz M., Altın C., Tekin A., Erol T., Arer I., Nursal T.Z., Törer N., Erol V., Müderrisoğlu H. (2018). Assessment of Atrial Fibrillation and Ventricular Arrhythmia Risk after Bariatric Surgery by P Wave/QT Interval Dispersion. Obes. Surg..

[57] Day C.P., McComb J.M., Campbell R.W. (1990). QT dispersion: An indication of arrhythmia risk in patients with long QT intervals. Br. Heart J..

[58] Perkiömäki J.S., Koistinen M.J., Yli-Mäyry S., Huikuri H.V. (1995). Dispersion of QT interval in patients with and without susceptibility to ventricular tachyarrhythmias after previous myocardial infarction. J. Am. Coll. Cardiol..

[59] Batchvarov V., Malik M. (2000). Measurement and interpretation of QT dispersion. Prog. Cardiovasc. Dis..

[60] Simson M.B., Untereker W.J., Spielman S.R., Luchi R. (1983). Relation between late potentials on the body surface and directly recorded fragmented electrograms in patients with ventricular tachycardia. Am. J. Cardiol..

[61] Berbari E.J., Vasquez C. (2010). Cardiac late potential signals and sources. J. Electrocardiol..

[62] Tuncer M., Gunes Y., Guntekin U., Gumrukcuoglu H.A., Eryonucu B., Guler N., Dilek I., Demir C. (2009). Heart rate variability in patients with iron deficiency anemia. Arq. Bras. Cardiol..

[63] Schneider S.A. (2016). Neurodegeneration with Brain Iron Accumulation. Curr. Neurol. Neurosci. Rep..

[64] O’Sullivan J.S., Lyne A., Vaughan C.J. (2021). COVID-19-induced postural orthostatic tachycardia syndrome treated with ivabradine. BMJ Case Rep..

[65] Pop-Busui R., Evans G.W., Gerstein H.C., Fonseca V., Fleg J.L., Hoogwerf B.J., Genuth S., Grimm R.H., Corson M.A., Prineas R. (2010). Effects of cardiac autonomic dysfunction on mortality risk in the Action to Control Cardiovascular Risk in Diabetes (ACCORD) trial. Diabetes Care.

[66] Carthy E.R. (2013). Autonomic dysfunction in essential hypertension: A systematic review. Ann. Med. Surg..

[67] Fudim M., Sobotka P.A., Dunlap M.E. (2021). Extracardiac Abnormalities of Preload Reserve: Mechanisms Underlying Exercise Limitation in Heart Failure with Preserved Ejection Fraction, Autonomic Dysfunction, and Liver Disease. Circ. Heart Fail..

[68] Petrie J.R., Guzik T.J., Touyz R.M. (2018). Diabetes, Hypertension, and Cardiovascular Disease: Clinical Insights and Vascular Mechanisms. Can. J. Cardiol..

[69] Ushakov A., Ivanchenko V., Gagarina A. (2023). Heart Failure And Type 2 Diabetes Mellitus: Neurohumoral, Histological And Molecular Interconnections. Curr. Cardiol. Rev..

[70] Muñoz M., García-Erce J.A., Remacha Á.F. (2011). Disorders of iron metabolism. Part II: Iron deficiency and iron overload. J. Clin. Pathol..

[71] Levi S., Taveggia C. (2014). Iron homeostasis in peripheral nervous system, still a black box?. Antioxid. Redox Signal..

[72] Obeagu G.U., Altraide B.O., Obeagu E.I. (2025). Iron deficiency anemia in pregnancy and related complications with specific insight in Rivers State, Nigeria: A narrative review. Ann. Med. Surg..

[73] Ando Y., Asahara K., Obayashi K., Suhr O., Yonemitsu M., Yamashita T., Tashima K., Uchino M., Ando M. (1996). Autonomic dysfunction and anemia in neurologic disorders. J. Auton. Nerv. Syst..

[74] Medigue C., Vermeiren M., Garrido M., Pena M., Peirano P. (1996). Assessment of autonomic dysfunction in iron-deficient anemic infants by cardio-respiratory demodulation. Proceedings of 18th Annual International Conference of the IEEE Engineering in Medicine and Biology Society, Amsterdam, The Netherlands, 31 October–3 November 1996.

[75] Nand N., Mohan R., Khosla S.N., Kumar P. (1989). Autonomic function tests in cases of chronic severe anaemia. J. Assoc. Physicians India.

[76] Touyz R.M., Camargo L.L. (2023). Reactive oxygen species and oxidative stress. Primer on the Autonomic Nervous System.

[77] Montezano A., Touyz R. (2012). Reactive Oxygen Species and the Cardiovascular System.

[78] Hirooka Y. (2011). Oxidative stress in the cardiovascular center has a pivotal role in the sympathetic activation in hypertension. Hypertens. Res..

[79] Jumrussirikul P., Dinerman J., Dawson T.M., Dawson V.L., Ekelund U., Georgakopoulos D., Schramm L.P., Calkins H., Snyder S.H., Hare J.M. (1998). Interaction between neuronal nitric oxide synthase and inhibitory G protein activity in heart rate regulation in conscious mice. J. Clin. Investig..

[80] Piccirillo G., Raffaele Q., Fimognari F., Moisè A., Mario M., Lionetti M., Naso C., Di Carlo S., Nocco M., Magrì D. (2004). Influence of L-arginine and vitamin C on the autonomic nervous system in chronic heart failure secondary to ischemic cardiomyopathy. Am. J. Cardiol..

[81] Lindley T.E., Doobay M.F., Sharma R.V., Davisson R.L. (2004). Superoxide is involved in the central nervous system activation and sympathoexcitation of myocardial infarction-induced heart failure. Circ. Res..

[82] Kardelen F., Tezcan G., Akcurin G., Ertug H., Yesilipek A. (2008). Heart rate variability in patients with thalassemia major. Pediatr. Cardiol..

[83] Stamboulis E., Vlachou N., Voumvourakis K., Andrikopoulou A., Arvaniti C., Tsivgoulis A., Athanasiadis D., Tsiodras S., Tentolouris N., Triantafyllidi H. (2012). Subclinical autonomic dysfunction in patients with β-thalassemia. Clin. Auton. Res..

[84] Salama M., El-Nemr S., Badraia I., Zoair A. (2020). P-Wave and QT Dispersion in Children with βeta-Thalassemia. J. Adv. Med. Med. Res..

[85] Cao A., Galanello R. (2010). Beta-thalassemia. Genet. Med..

[86] Origa R., Galanello R., Perseu L., Tavazzi D., Domenica Cappellini M., Terenzani L., Forni G.L., Quarta G., Boetti T., Piga A. (2009). Cholelithiasis in thalassemia major. Eur. J. Haematol..

[87] Franzoni F., Galetta F., Di Muro C., Buti G., Pentimone F., Santoro G. (2004). Heart rate variability and ventricular late potentials in beta-thalassemia major. Haematologica.

[88] Santangeli P., Infusino F., Sgueglia G.A., Sestito A., Lanza G.A. (2008). Ventricular late potentials: A critical overview and current applications. J. Electrocardiol..

[89] Elming H., Holm E., Jun L., Torp-Pedersen C., Køber L., Kircshoff M., Malik M., Camm J. (1998). The prognostic value of the QT interval and QT interval dispersion in all-cause and cardiac mortality and morbidity in a population of Danish citizens. Eur. Heart J..

[90] Kautzner J., Pedersen A.K., Peichl P. (2016). Electro-Anatomical Mapping of the Heart: An Illustrated Guide to the Use of the CARTO System.

[91] Pérez-Riera A.R., de Abreu L.C., Barbosa-Barros R., Grindler J., Fernandes-Cardoso A., Baranchuk A. (2016). P-wave dispersion: An update. Indian Pacing Electrophysiol. J..

[92] Seravalle G., Lonati L., Buzzi S., Cairo M., Trevano F.Q., Dell’oRo R., Facchetti R., Mancia G., Grassi G. (2015). Sympathetic nerve traffic and baroreflex function in optimal, normal, and high-normal blood pressure states. J. Hypertens..

[93] Li C.X., Zhang L., Wang P., Sun L. (2019). Clinicopathological diagnosis and treatment of juvenile hemochromatosis. Chin. Med. J..

[94] Tank J., Diedrich A., Szczech E., Luft F.C., Jordan J. (2005). Baroreflex regulation of heart rate and sympathetic vasomotor tone in women and men. Hypertension.

[95] McDaid C., Duree K.H., Griffin S.C., Weatherly H.L., Stradling J.R., Davies R.J., Sculpher M.J., Westwood M.E. (2009). A systematic review of continuous positive airway pressure for obstructive sleep apnoea–hypopnoea syndrome. Sleep Med. Rev..

[96] Ferini-Strambi L., Walters A.S., Sica D. (2014). The relationship among restless legs syndrome (Willis-Ekbom Disease), hypertension, cardiovascular disease, and cerebrovascular disease. J. Neurol..

[97] Beck-da-Silva L., Piardi D., Soder S., Rohde L.E., Pereira-Barretto A.C., de Albuquerque D., Bocchi E., Vilas-Boas F., Moura L.Z., Montera M.W. (2013). IRON-HF study: A randomized trial to assess the effects of iron in heart failure patients with anemia. Int. J. Cardiol..

[98] Bekfani T., Pellicori P., Morris D., Ebner N., Valentova M., Sandek A., Doehner W., Cleland J.G., Lainscak M., Schulze P.C. (2019). Iron deficiency in patients with heart failure with preserved ejection fraction and its association with reduced exercise capacity, muscle strength and quality of life. Clin. Res. Cardiol..

[99] Sindone A.P., Haikerwal D., Audehm R.G., Neville A.M., Lim K., Parsons R.W., Piazza P., Liew D. (2021). Clinical characteristics of people with heart failure in Australian general practice: Results from a retrospective cohort study. ESC Heart Fail..

[100] Pezel T., Audureau E., Mansourati J., Baudry G., Ben Driss A., Durup F., Fertin M., Godreuil C., Jeanneteau J., Kloeckner M. (2021). Diagnosis and treatment of iron deficiency in heart failure: oFICSel study by the French heart failure working group. ESC Heart Fail..

[101] Beale A., Carballo D., Stirnemann J., Garin N., Agoritsas T., Serratrice J., Kaye D., Meyer P., Carballo S. (2019). Iron deficiency in acute decompensated heart failure. J. Clin. Med..

[102] Ponikowski P., van Veldhuisen D.J., Comin-Colet J., Ertl G., Komajda M., Mareev V., McDonagh T., Parkhomenko A., Tavazzi L., Levesque V. (2015). Beneficial effects of long-term intravenous iron therapy with ferric carboxymaltose in patients with symptomatic heart failure and iron deficiency†. Eur. Heart J..

[103] Armony-Sivan R., Eidelman A.I., Dror G., Sagi A. (2004). Iron deficiency anemia and autonomic regulation in infancy. Dev. Psychobiol..

[104] Georgieff M.K. (2011). Long-term brain and behavioral consequences of early iron deficiency. Nutr. Rev..

[105] Kolkiran A., Tutar E., Atalay S., Deda G., Cin Ş. (2005). Autonomic nervous system functions in children with breath-holding spells and effects of iron deficiency. Acta Paediatr..

[106] Martins Wde A., Lopes H.F., Consolim-Colombo F.M., Gualandro S.d.F.M., Arteaga-Fernández E., Mady C. (2012). Cardiovascular autonomic dysfunction in sickle cell anemia. Auton. Neurosci..

[107] Wijarnpreecha K., Kumfu S., Chattipakorn S.C., Chattipakorn N. (2015). Cardiomyopathy associated with iron overload: How does iron enter myocytes and what are the implications for pharmacological therapy?. Hemoglobin.

[108] Seravalle G., Piperno A., Mariani R., Pelloni I., Facchetti R., Dell’ORo R., Cuspidi C., Mancia G., Grassi G. (2016). Alterations in sympathetic nerve traffic in genetic haemochromatosis before and after iron depletion therapy: A microneurographic study. Eur. Heart J..

[109] Yuksel I.O., Koklu E., Kurtoglu E., Arslan S., Cagirci G., Karakus V., Kus G., Cay S., Kucukseymen S. (2016). The Association between Serum Ferritin Level, Tissue Doppler Echocardiography, Cardiac T2* MRI, and Heart Rate Recovery in Patients with Beta Thalassemia Major. Acta Cardiol. Sin..

