# Supplementary material for: From Iron Deficiency to Overload: A Missing Link in the Mechanisms of Cardiac Autonomic Nervous System Dysfunction
Source: J Clin Med. 2026 Feb 28;15(5):1871. doi: 10.3390/jcm15051871 (PMC12986322; doi:10.3390/jcm15051871)
Supplement: Supplementary file 1 [file jcm-15-01871-s001.zip › jcm-4125840-supplementary.pdf]

Table S1. Full Search Strategies for All Databases

Database	Date of Search	Search Strategy	Filters Applied	Records Retrieved
**PubMed (MEDLINE)**	12 January 2025	("Autonomic Nervous System"[Mesh] OR "Autonomic Nervous System Diseases"[Mesh] OR "autonomic nervous system dysfunction"[Title/Abstract] OR "autonomic dysfunction"[Title/Abstract] OR dysautonomia[Title/Abstract] OR "heart rate variability"[Title/Abstract] OR HRV[Title/Abstract] OR baroreflex[Title/Abstract]) AND ("Iron Metabolism Disorders"[Mesh] OR "Iron Deficiency"[Mesh] OR "Iron-Deficiency Anemia"[Mesh] OR "Iron Overload"[Mesh] OR "Hemochromatosis"[Mesh] OR "beta-Thalassemia"[Mesh] OR "Anemia, Sickle Cell"[Mesh] OR "iron metabolism disorder*"[Title/Abstract] OR "iron deficiency"[Title/Abstract] OR "iron deficiency anemia"[Title/Abstract] OR "iron overload"[Title/Abstract] OR "hereditary hemochromatosis"[Title/Abstract] OR "beta-thalassemia"[Title/Abstract] OR "sickle cell anemia"[Title/Abstract])	English language; published between 1983 and 2025	[152]
**Scopus**	12 January 2025	TITLE-ABS-KEY("autonomic nervous system dysfunction" OR "autonomic dysfunction" OR dysautonomia OR "heart rate variability" OR HRV OR baroreflex) AND TITLE-ABS-KEY("iron metabolism disorder*" OR "iron deficiency" OR "iron deficiency anemia" OR "iron overload" OR "hereditary hemochromatosis" OR "beta-thalassemia" OR "sickle cell anemia")	English language; Article; published between 1983 and 2025	[209]
**Wiley Online Library**	12 January 2025	("autonomic nervous system dysfunction" OR "autonomic dysfunction" OR dysautonomia OR "heart rate variability" OR HRV OR baroreflex) AND ("iron metabolism disorder*" OR "iron deficiency" OR "iron deficiency anemia" OR "iron overload" OR "hereditary hemochromatosis" OR "beta-thalassemia" OR "sickle cell anemia")	Research Articles; English; published between 1983 and 2025	[1527]
